# Correction: Lipid phosphate phosphatases dimerise, but this interaction is not required for in vivo activity

**DOI:** 10.1186/1471-2091-6-3

**Published:** 2005-03-03

**Authors:** C Burnett, P Makridou, L Hewlett, K Howard

**Affiliations:** 1 Department of Physiology, MRC Laboratory for Molecular Cell Biology, University College London, Gower St, London WC1E 6BT, UK

After the publication of this work [1] it was brought to our attention that the concentrations of the reagents listed for the cell lysis buffer are those of the original *stock *solutions and not the required final concentrations. The correct final concentrations for this buffer are as follows:

HEPES 50 mM

NaCl 100 mM

NaF 10 mM

EDTA 5 mM

Na3VO4 0.5 mM

NEM 2 mM

Triton 0.1%

Complete protease inhibitors (Roche).

We regret any inconvenience that this inaccuracy may have caused, and thank Dr. Bräuer for bringing it to our attention.
